# Procalcitonin to guide duration of antibiotic therapy in intensive care patients: a randomized prospective controlled trial

**DOI:** 10.1186/cc7903

**Published:** 2009-06-03

**Authors:** Marcel Hochreiter, Thomas Köhler, Anna Maria Schweiger, Fritz Sixtus Keck, Berthold Bein, Tilman von Spiegel, Stefan Schroeder

**Affiliations:** 1 Department of Anesthesiology and Intensive Care Medicine, West Coast Hospital, Esmarchstrasse 50, 25746 Heide, Germany; 2 Department of Laboratory Medicine and Clinical Chemistry, West Coast Hospital, Esmarchstrasse 50, 25746 Heide, Germany; 3 Medical Clinic, West Coast Hospital, Esmarchstr. 50, 25746 Heide, Germany; 4 Department of Anesthesiology and Intensive Care Medicine, University Hospital Schleswig-Holstein, Campus Kiel, Schwanenweg 21, 24105 Kiel, Germany

## Abstract

**Introduction:**

The development of resistance by bacterial species is a compelling issue to reconsider indications and administration of antibiotic treatment. Adequate indications and duration of therapy are particularly important for the use of highly potent substances in the intensive care setting. Until recently, no laboratory marker has been available to differentiate bacterial infection from viral or non-infectious inflammatory reaction; however, over the past years, procalcitonin (PCT) is the first among a large array of inflammatory variables that offers this possibility. The present study aimed to investigate the clinical usefulness of PCT for guiding antibiotic therapy in surgical intensive care patients.

**Methods:**

All patients requiring antibiotic therapy based on confirmed or highly suspected bacterial infections and at least two concomitant systemic inflammatory response syndrome criteria were eligible. Patients were randomly assigned to either a PCT-guided (study group) or a standard (control group) antibiotic regimen. Antibiotic therapy in the PCT-guided group was discontinued, if clinical signs and symptoms of infection improved and PCT decreased to <1 ng/ml or the PCT value was >1 ng/ml, but had dropped to 25 to 35% of the initial value over three days. In the control group antibiotic treatment was applied as standard regimen over eight days.

**Results:**

A total of 110 surgical intensive care patients receiving antibiotic therapy after confirmed or high-grade suspected infections were enrolled in this study. In 57 patients antibiotic therapy was guided by daily PCT and clinical assessment and adjusted accordingly. The control group comprised 53 patients with a standardized duration of antibiotic therapy over eight days. Demographic and clinical data were comparable in both groups. However, in the PCT group the duration of antibiotic therapy was significantly shorter than compared to controls (5.9 +/- 1.7 versus 7.9 +/- 0.5 days, *P *< 0.001) without negative effects on clinical outcome.

**Conclusions:**

Monitoring of PCT is a helpful tool for guiding antibiotic treatment in surgical intensive care patients. This may contribute to an optimized antibiotic regimen with beneficial effects on microbial resistance and costs in intensive care medicine.

**Annotation:**

Results were previously published in German in Anaesthesist 2008; 57: 571–577 (PMID: 18463831).

**Trial registration:**

ISRCTN10288268

## Introduction

Early differentiation between sepsis and systemic inflammatory response syndrome (SIRS) is of central importance for therapeutic decision-making. Although patients with SIRS will not require antibiotic therapy, immediate administration of antibiotics is essential for improved survival in patients with sepsis [1]. Despite apparently clear sepsis definitions [2], early differentiation between SIRS and sepsis is often difficult in clinical practice. SIRS criteria such as fever, tachycardia, and tachypnoea, are observed in most intensive care patients and are fairly nonspecific features of various underlying diseases. On the other hand, fever and leukocytosis may not necessarily be present in clinically manifest sepsis [3,4]. Another problem for the diagnosis of sepsis is the evidence of infection. In a prospective study of 300 hospital patients with fever (>38.0°C), Bossink and colleagues [5] were able to demonstrate that at least two SIRS criteria were present in 95% of these patients and clinical suspicion of sepsis in 71%, but only 44% had microbiologic proven infection.

Besides profound clinical experience there is an urgent need for biomarkers, which allow early differentiation between SIRS and sepsis. Currently, procalcitonin (PCT) has emerged as a laboratory variable that meets this demand [6]. Elevated PCT values indicate systemic bacterial infections with high sensitivity. In their studies, Harbarth and colleagues [7] and Oberhoffer and colleagues [3] confirmed that monitoring of PCT reliably differentiates SIRS and sepsis. Moreover, it was recently shown, that PCT is a valuable tool to guide antibiotic treatment in medical patients with pulmonary diseases [6,8,9]. However, there is no evidence in the literature about whether this applies to antibiotic treatment in intensive care patients with severe infections as well. In clinical practice, treatment intervals of between 10 and 14 days are favored [10]. Unnecessarily long antibiotic administration is not only expensive, it also leads to increased complications. Drug-induced side effects, such as allergic reactions, antibiotic-associated colitis, and the risk of life-threatening infections from multi-resistant bacteria, significantly rise [11-13]. The causal relationship between antibiotic use and antibiotic resistance has been well established [14]. The frequency of multi-resistant pathogens in patients undergoing long-term antibiotic therapy is clearly increased [11]. In this context, newly arising fungal infections in seven-day antibiotic treatment have become of great concern, even in immunocompetent patients [15].

Therefore, we aimed to address the role of daily PCT-serum determinations for guiding the length of antibiotic treatment in surgical intensive care patients in the present randomized trial.

## Materials and methods

Ethics commission approval was obtained from the Medical Faculty at Christian Albrecht University of Kiel (A158/05) for our trial in the surgical intensive care ward at the West Coast Hospital Heide and written informed consent was obtained from each individual. All patients requiring antibiotic therapy based on confirmed or highly suspected bacterial infections and at least two concomitant SIRS criteria were eligible [2].

Patients were randomly assigned to either a PCT-guided (study group) or a standard (control group) antibiotic regimen. For both groups, antibiotics were selected based on confirmed or highly suspected bacterial infections. The type of antibiotic substance chosen was either calculated according to the expected microbiologic spectrum and/or adjusted to the isolated organisms whenever possible. IL-6, C-reactive protein (CRP), PCT, and leukocyte count were determined daily for each group on a routine laboratory basis. In addition, Sequential Organ Failure Assessment (SOFA) scores were calculated daily [16] to stratify and monitor disease severity.

Antibiotic therapy in the PCT-guided group was discontinued if clinical signs and symptoms of infection improved and PCT decreased to less than 1 ng/ml, or if the PCT value was more than 1 ng/ml, but had dropped to 25 to 35% of the initial value over three days. In the control group, antibiotic treatment was applied as standard regimen over eight days. Irrespective of the study group and at any time point, the physician in charge had the option to proceed with or adjust the antibiotic treatment, if there were clinical reasons to do so.

IL-6 was measured by using the ACCESS^® ^Immunoassay (Beckman Coulter GmbH, Krefeld, Germany) and CRP was measured by using the Vitros Chemistry System^® ^5.1 FF (Ortho-Clinical Diagnostics GmbH, Neckargemünd, Germany) according to the manufacturers' instructions. The leukocyte count was analyzed with the Sysmex Hematology Device (Sysmex Deutschland GmbH, Norderstedt, Germany). The BRAHMS PCT LIA^® ^(BRAHMS Aktiengesellschaft, Hennigsdorf, Germany) was used for the PCT determinations according to manufacturers' protocols. The reference values in healthy subjects for the aforementioned laboratory variables are as follows: IL-6 less than 15 pg/ml, CRP less than 0.7 mg/dl, PCT less than 0.5 ng/ml, and leukocytes 4 to 10 × 10^3^/μl.

Clinical parameters such as age, gender, underlying diagnoses, and the Simplified Acute Physiology Score (SAPS) II for stratification of disease severity were documented in all patients upon study inclusion. Throughout the study, the duration of intensive care stay and antibiotic treatment, all antibiotic substance classes administered, and the outcome were recorded.

Except when otherwise stated, continuous variables are presented as mean value and standard deviation for descriptive statistics. Comparative statistics were performed by using the Mann-Whitney-Wilcoxon U test. For comparison of proportions (gender, diagnosis, antibiotic substance classes, survival/death) the chi-squared test was employed. A *P *< 0.05 was considered statistically significant.

## Results

Of 395 patients screened, a total of 110 patients fulfilling the inclusion criteria were entered in the study from January 2006 to March 2007. In these patients, a minimum of two SIRS criteria were present at the start of antibiotic therapy due to a confirmed or highly suspected bacterial infection. Patients who refused study consent, whose antibiotic treatment had been initiated before intensive care admission, or who had therapy limitations were excluded from the study. Fifty-seven patients were randomly assigned to the PCT-guided group and 53 to the control group.

In the present prospective, randomised open study, both treatment groups were comparable in terms of age, gender distribution, diagnoses, disease severity as reflected by SAPS II, and outcome (Table 1). The distribution of antibiotic classes used was comparable as well (Table 2). The duration of antibiotic treatment in the PCT-guided group was with 5.9 ± 1.7 days significantly shorter than in the control group (*P *< 0.001) with 7.9 ± 0.5 days without any negative effects on treatment success (Tables 1 and 2). In addition, the length of intensive care treatment in the PCT-guided group was 15.5 ± 12.5 days and significantly shorter than that in the control group with 17.7 ± 10.1 days (*P *= 0.046; Table 1).

**Table 1:** Demographic and clinical data

	**Controls**	**PCT-guided antibiotic therapy**	***P***
**Patients (n)**	53	57	
**Age (years)**	66.6 ± 15.5	67.3 ± 14.4	>0.05
			
**Gender**			>0.05
Male	29	29	
Female	24	28	
			
**Diagnoses**			>0.05
Pneumonia	19	24	
Peritonitis	30	29	
Soft tissue infection	1	2	
Urosepsis	3	2	
			
**SAPS II**	40.5 ± 15.1	40.1 ± 17.1	>0.05
			
**Intensive care (days)**	17.7 ± 10.1	15.5 ± 12.5	0.046
**Hospital discharge**			>0.05
Survived	39	42	
Deceased	14	15	

PCT = procalcitonin; SAPS II = Simplified Acute Physiology Score II. Mean ± standard deviation.

**Table 2:** Frequency of used antibiotics and length of therapy

	**Controls**	**PCT-guided antibiotic therapy**	***P***
**Antibiotic classes (%)**			>0.05
Acylaminopenicillin + BLI	51.8	55.2	
Acylaminopenicillin + nitroimidazole	19.6	15.5	
Carbapenem	8.8	10.4	
Aminobenzylpenicillin + BLI	5.4	6.9	
Fluorochinolone	5.4	5.2	
Cephalosporins of Group 3b	5.4	3.4	
Others	3.6	3.4	
**Length of antibiotic therapy (days)**	7.9 ± 0.5	5.9 ± 1.7	<0.001

BLI = β-lactamase inhibitor; PCT = procalcitonin. Mean ± standard deviation.

**Figure 1:**
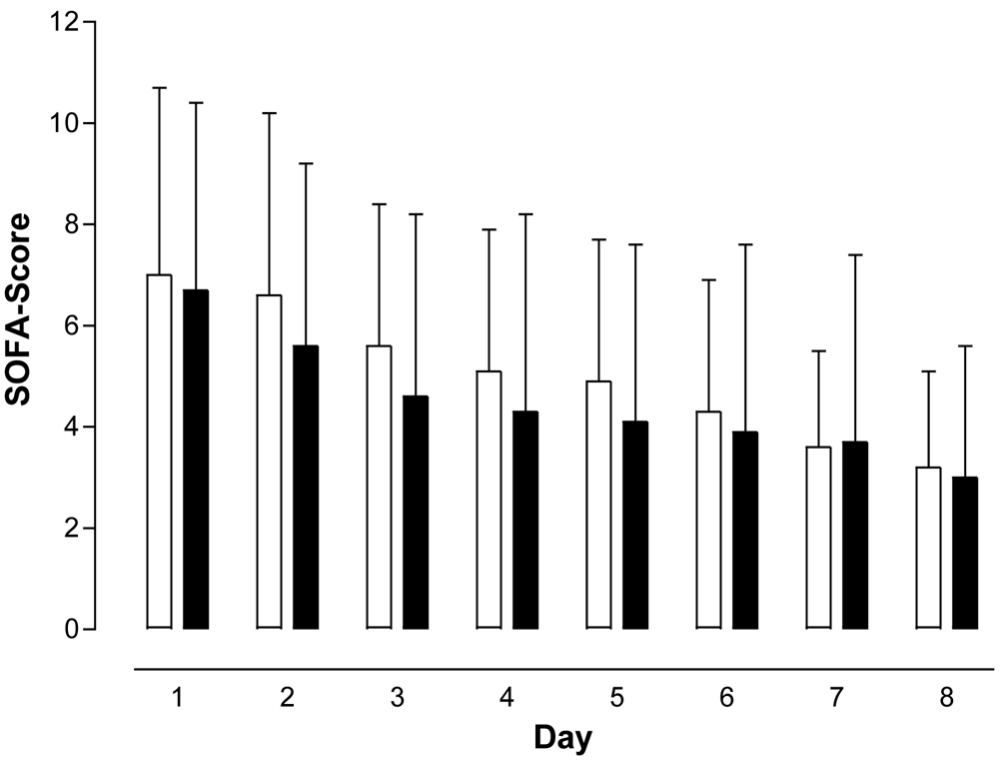
Sequential Organ Failure Assessment scores. No difference in score was seen between patients with procalcitonin-guided antibiotic treatment (filled columns) and the control group (empty columns). Mean ± standard deviation. SOFA = Sequential Organ Failure Assessment.

**Figure 2:**
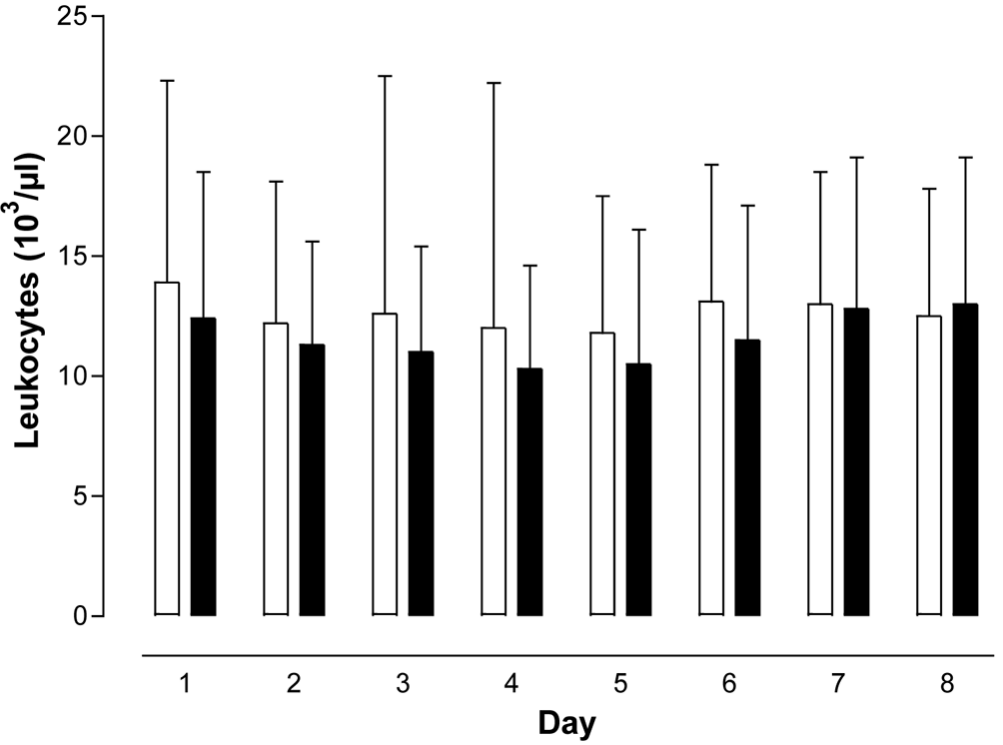
Leukocyte values. No difference in values was seen between patients with procalcitonin-guided antibiotic treatment (filled columns) and the control group (empty columns). Mean ± standard deviation.

**Figure 3:**
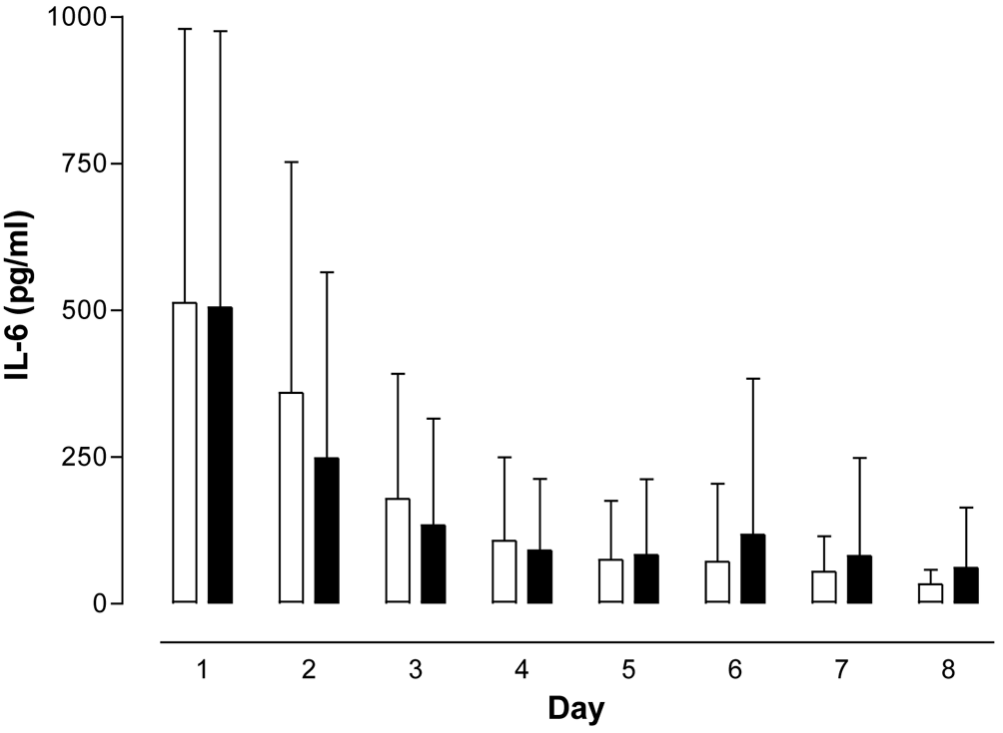
IL-6 concentrations. No difference in concentration was seen between patients with procalcitonin-guided antibiotic treatment (filled columns) and the control group (empty columns). Mean ± standard deviation.

**Figure 4:**
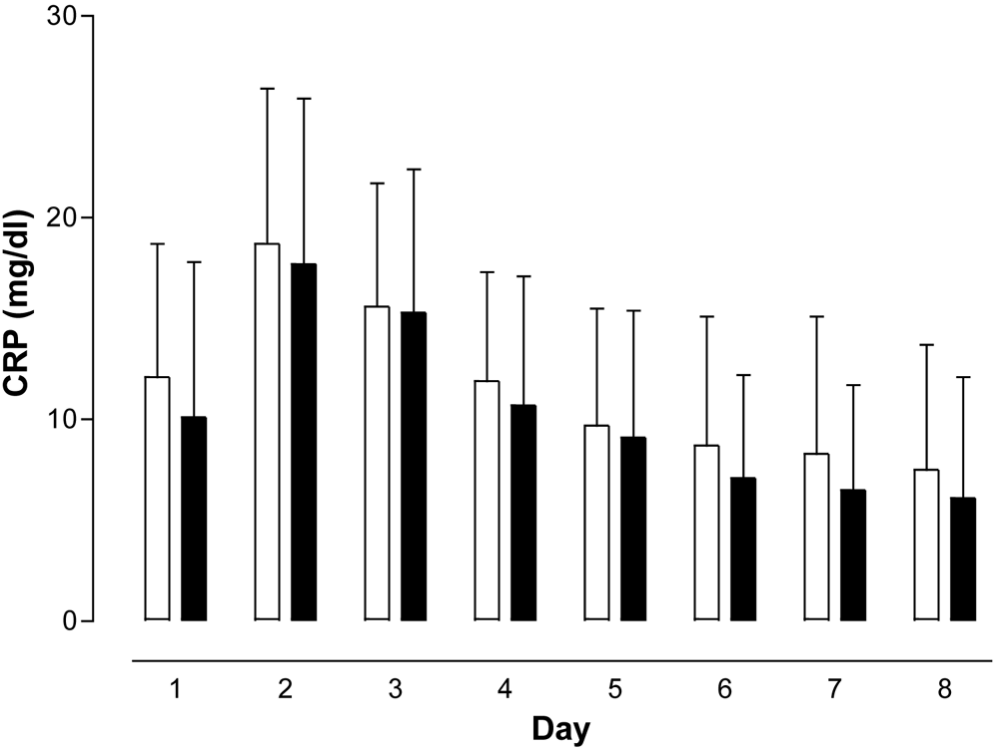
C-reactive protein concentrations. No difference in concentration was seen between patients with procalcitonin-guided antibiotic treatment (filled columns) and the control group (empty columns). Mean ± standard deviation. CRP = C-reactive protein.

**Figure 5:**
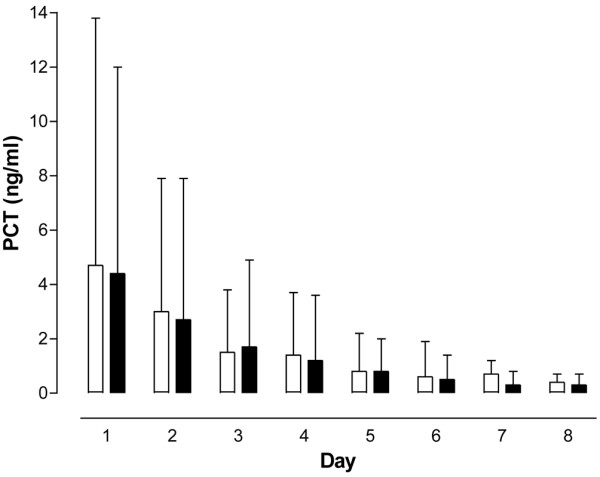
Procalcitonin concentrations. No difference in concentration was seen between patients with procalcitonin (PCT)-guided antibiotic treatment (filled columns) and the control group (empty columns). Mean ± standard deviation.

**Figure 6:**
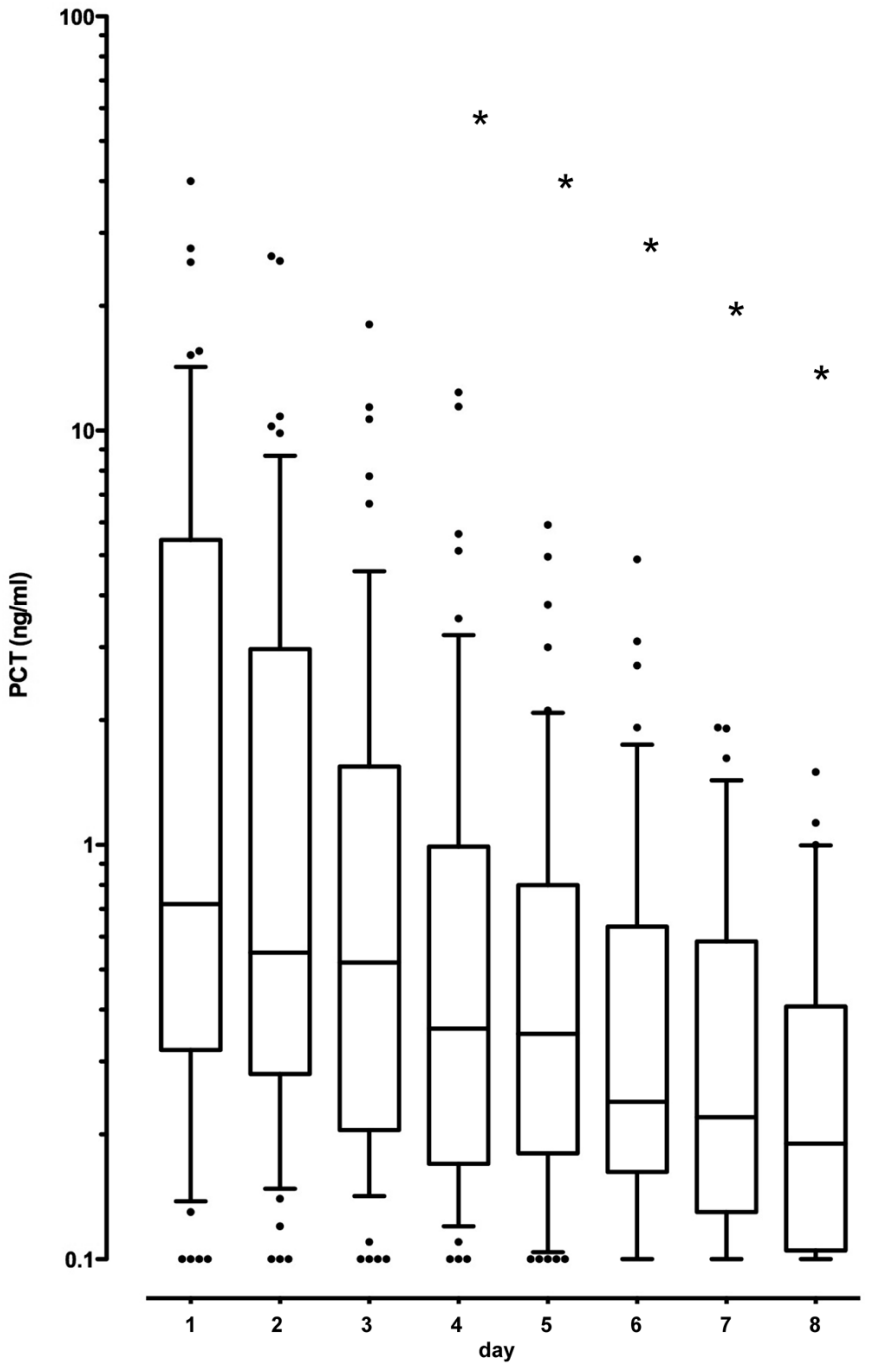
Procalcitonin levels solely shown for the intervention arm. In the group with procalcitonin (PCT)-guided antibiotic therapy duration, the PCT values starting on the fourth day are significantly lower in comparison to the initial value (box-plots with lower quartile, median and upper quartile, 0.1- and 0.9-quantile for the whisker length and outliers as item representation). • = Outliers, *P < 0.05.

The SOFA score as a means to assess disease severity in intensive care patients, the leukocyte count, as well as IL-6, CRP, and PCT concentrations did not differ between the PCT-guided and the control antibiotic treatment groups at any of the time points the respective parameters were analyzed (Figures 1, 2, 3, 4 and 5). The only difference observed was in the PCT-guided group, which revealed significantly lower PCT values compared with the initial values starting from the fourth day of the study (Figure 6).

## Discussion

Despite being considered as surrogate signs of systemic inflammation, SIRS criteria are not always the result of a systemic infection. They may arise due to general impairment of the human organism [17]. Standard laboratory variables such as CRP have a slow kinetic profile rendering it an inappropriate marker for a fast evaluation of the dynamics of an infection [18]. The same applies for the leukocyte count [3]. Furthermore, IL-6 levels are not indicators of infections or sepsis, although they correlate well with the degree of severity of inflammation [7].

Sepsis continues to be the leading cause of morbidity and in its severe form (severe sepsis, septic shock, and multi-organ dysfunction) of mortality in intensive care patients [19]. Treatment is generally most effective if it is initiated without delay according to current recommendations and guidelines. Therefore, a fast and accurate diagnosis of infection and sepsis is of central importance.

Harbarth and colleagues have convincingly shown that PCT values differentiate patients with SIRS from those with sepsis. Moreover, both PCT and IL-6 correlate with the systemic severity of inflammatory response [7]. In the same study the authors demonstrated that PCT rapidly decreased to reference values in successfully treated patients, whereas patients with SIRS in the absence of infection revealed PCT values less than 1 ng/ml throughout the clinical course. More recent studies suggest that a PCT-based algorithm significantly shortens the length of antibiotic therapy without affecting the treatment success and outcome [8,9].

Based on these data we hypothesized that the length of antibiotic therapy can be optimized and shortened in surgical intensive care patients as well with daily PCT guidance. To date, there is no evidence in the literature for this approach in a surgical intensive care setting. In a study by Chastre and colleagues [11], 8-day and 15-day antibiotic therapy in patients with respiratory-associated pneumonia and systemic sepsis were compared. The authors did not observe any difference between both groups in terms of treatment success. However, the rate of recurrent infections with *Pseudomonas aeruginosa *was greater for the eight-day treatment group, whereas a markedly higher incidence of multi-resistant pathogens was found in 60% of the reinfections in the 15-day treatment group. Micek and colleagues [20] also studied the length of antibiotic therapy in respiratory associated pneumonia and postulated a therapeutic interval of seven to eight days as sufficient in responding patients. A prospective cluster-randomized study by Christ-Crain and Müller [8] in more than 300 patients with community acquired pneumonia could show that antibiotic treatment was reduced from 12 to 5 days by applying a PCT-based algorithm. Accordingly, Nobre and colleagues [9] could shorten the length of antibiotic therapy in patients with sepsis using a PCT-based algorithm from a median of 10 to 6 days without any influence on treatment outcome. Based on the literature and study results on PCT-guidance in intensive care patients with infections, a maximum duration of antibiotic therapy of eight days appears to be sufficient and safe.

The challenge of intensive care medicine is clearly the early differentiation of patients with SIRS from those with sepsis. Although this is mainly based on clinical course and symptoms, biochemical inflammation and sepsis markers are often indispensable to establish diagnosis. However, despite these limitations, a delayed start of antibiotic therapy should be avoided whenever possible. A study from the USA was able to demonstrate that patients with ventilator-associated pneumonia who received antibiotic therapy only 24 hours after established diagnosis, exhibited a seven times higher mortality rate compared with patients started on adequate therapy earlier [21]. Accordingly, Kumar and colleagues [1] could show that the hospital mortality rate for patients with septic shock increased by about 7% per hour within the first six hours of delayed antibiotic administration.

The increasing resistance to standard antibiotics has driven the need to revise treatment recommendations in terms of diagnoses and duration of therapy. Current data have impressively shown a dramatic increase of resistance against antibiotic groups by infectious micro-organisms [22]. Evidence from the literature suggests that antibiotic therapy significantly increases the risk of fungal infections, if administered for more than seven days [15] and significantly increases the development of resistance if applied for more than 10 days [10]. One of the main reasons for the increasing rate of resistance is non-controlled antibiotic use and long-term treatment in intensive care wards [14,23]. The development of resistance is strongly dependent on the antibiotic substance classes used, as well as on the bacterium itself, and can last between one day and three decades. Once resistance has emerged it is only slowly or not reversible, even after changes in the selection pressure [14]. Therefore, it is postulated that both the early decision for or against antibiotic therapy as well as a continuous re-evaluation of the neccessity of anti-microbial therapy have a favorable influence on this development. Singh and colleagues [12] investigated intensive care patients with pneumonia and found that discontinuing antibiotic treatment after three days in the absence of a suspected infection did not worsen outcome. This approach could reduce the development of resistance. During the course of disease and treatment PCT-guided algorithms can help to shorten the length of antibiotic therapy without any unfavorable effects on treatment success and outcome. This is well-documented in the literature and by our own results [8,9].

Beyond a reduction of the length of antibiotic treatment PCT guidance also had a favorable effect on the length of the intensive care stay. These findings are in accordance with a recent publication by Nobre and colleagues [9] who observed a reduction of the length of antibiotic therapy along with a two-day shorter average duration of intensive care treatment using a PCT-based algorithm.

As already mentioned before, delayed diagnosis and inadequate antibiotic therapy have unfavorable consequences for the success of treatment in patients with sepsis [1,11-13,15]. On the other hand, if antibiotic treatment is inadequate and too long the development of antibiotic resistance is favoured [11,14]. The duration of antibiotic therapy is based on the type of infection, suspected or proven pathogens, and the clinical course with potential recurrence of clinical signs and symptoms of infection. The length of treatment should be kept as short as possible [10].

## Conclusions

PCT assessment provides a helpful tool to decide on the duration of antibiotic treatment, if interpreted in the clinical context including the underlying disease. This can substantially improve to determine adequate duration of antibiotic therapy with favorable effects on resistance and intensive care costs.

## Key messages

• PCT is the first laboratory marker among a large array of inflammatory variables that offers the possibility to differentiate bacterial infection from viral or non-infectious inflammatory reaction.

• PCT assessment provides a helpful tool to decide on the duration of antibiotic treatment, if interpreted in the clinical context including the underlying disease.

• PCT-based algorithm supports the cautious use of antibiotics and has a favourable effect on the clinical outcome.

• PCT-based algorithm is certainly practicable and simple.

• PCT-controlled antibiotic therapy must still be tested in heterogenous groups of patients, particularly for safety.

## Abbreviations

CRP: C-reactive protein; IL-6: interleukin-6; PCT: procalcitonin; SAPS: Simplified Acute Physiology Score; SIRS: systemic inflammatory response syndrome; SOFA: Sequential Organ Failure Assessment.

## Competing interests

SS has served as consultant and has received payments from BRAHMS AG for speaking engagements. All other authors declare no conflicts of interest.

## Authors' contributions

MH and SS conceived and designed the study, contributed to acquisition, analysis and interpretation of data, performed the statistical analysis, and drafted the manuscript. TK and AMS made substantial contributions to data acquisition and interpretation, and revised the manuscript. BB and FSK contributed to study design and revised the manuscript. TvS contributed substantially in all parts of the study and revised the manuscript.
